# Supplementary material for: HPV Infection of the Oropharyngeal, Genital and Anal Mucosa and Associated Dysplasia in People Living with HIV
Source: Viruses. 2023 May 15;15(5):1170. doi: 10.3390/v15051170 (PMC10222174; doi:10.3390/v15051170)
Supplement: Supplementary file 1 [file viruses-15-01170-s001.zip › viruses-2346921-supplementary.pdf]

Supplementary Table S1. Current HPV infection and presence of female genital dysplasia

	N=62
HPV+ PCR, n (%)	31 (50)
Genotype identification, n (%)	56 (90.3)
HPV-HR, n (%)	14 (25.0)
Number of HPV-HR serotypes, median (IQR)	0 (0-0.5)
HPV-LR, n (%)	17 (30.4)
Number of HPV-LR serotypes, median (IQR)	0 (0-1)
LR- and HR-HPV coinfection, n (%)	7 (12.5)
HPV 6, n (%)	2 (3.6)
HPV 16, n (%)	4 (7.1)
HPV 31, n (%)	1 (1.8)
HPV 35, n (%)	2 (3.6)
HPV 39, n (%)	1 (1.8)
HPV 40, n (%)	1 (1.8)
HPV 42, n (%)	4 (7.1)
HPV 43, n (%)	2 (3.6)
HPV 44, n (%)	4 (7.1)
HPV 44/55, n (%)	3 (5.4)
HPV 51, n (%)	5 (8.9)
HPV 54, n (%)	1 (1.8)
HPV 59, n (%)	3 (5.4)
HPV 61, n (%)	1 (1.8)
HPV 62, n (%)	1 (1.8)
HPV 62/81, n (%)	5 (8.9)
HPV 68, n (%)	2 (3.6)
HPV 72, n (%)	1 (1.8)
HPV 73, n (%)	2 (3.6)
HPV 82, n (%)	1 (1.8)
Simultaneous oral and genital HPV, n (%)	3 (5.2)
Cervical Cytology, n (%)	
-Normal	55 (88.7)

-LSIL	3 (1.0)
-ASCUS	4 (1.3)
Gynecological examination, n (%)	1 (1.7)
-CIN 1	1 (100)

IQR, interquartile range; PCR, polymerase chain reaction; HPV, human papillomavirus; LSIL, low-grade intraepithelial lesion; HSIL, high-grade intraepithelial lesion; ASCUS, atypical squamous cells of uncertain significance; CIN, cervical intraepithelial neoplasm.

Supplementary Table S2. Current HPV infection and the presence of anal dysplasia

	**N=288**
HPV+ PCR, n (%)	238 (82.6)
Identification of genotypes, n (%)	281 (97.5%)
HPV-HR, n (%)	189 (67.3)
Number of HPV-HR, median (IQR)	1(0-2)
HPV-LR, n (%)	168 (59.8)
Number of HPV-LR, median (IQR)	1 (0-2)
HR- and LR-HPV coinfection, n (%)	123 (43.8)
HPV 6	33 (11.7)
HPV 11	16 (5.7)
HPV 16	42 (14.9)
HPV 18	21 (7.5)
HPV 26	1 (0.4)
HPV 31	36 (12.8)
HPV 32	1 (0.4)
HPV 33	22 (7.8)
HPV 35	26 (9.3)
HPV 39	29 (10.3)
HPV 40	12 (4.3)
HPV 42	28 (10.0)
HPV 43	16 (5.7)
HPV 44	1 (0.4)
HPV 44/55	54 (19.2)
HPV 45	22 (7.8)

HPV 51	23 (8.2)
HPV 52	33 (11.7)
HPV 53	12 (4.3)
HPV 54	12 (4.3)
HPV 56	24 (8.5)
HPV 58	23 (8.2)
HPV 59	33 (11.7)
HPV 61	24 (8.5)
HPV 62/81	54 (19.2)
HPV 66	18 (6.4)
HPV 67	13 (4.6)
HPV 68	41 (14.6)
HPV 69	4 (1.4)
HPV 70	27 (9.6)
HPV 71	2 (0.7)
HPV 72	24 (8.5)
HPV 73	21 (7.5)
HPV 82	12 (4.3)
HPV 84	12 (4.3)
Simultaneous oral and anal HPV infection	25 (8.9)
	N =300
**Anal Cytology**, n (%)	
-Normal	118 (39.6)
-LSIL	110 (36.9)
-HSIL	8 (2.7)
-ASCUS	33 (11.1)
-Not valid	29 (9.7)
**HRA**, n (%)	89 (30.9)
- LSIL (AIN 1)	84 (29.2)
-HSIL (AIN 2-3 or carcinoma in situ)	5 (1.7)

IQR, interquartile range; PCR, polymerase chain reaction; HPV, human papillomavirus; LSIL, low-grade squamous intraepithelial lesion; HSIL, high-grade squamous intraepithelial lesion; ASCUS, atypical squamous cells of uncertain significance; HRA: high resolution anoscopy; AIN, anal intraepithelial neoplasm.
